# Re-defining standards of care in resectable head and neck squamous cell carcinoma—lessons from KEYNOTE-689 and NIVOPOSTOP

**DOI:** 10.1093/oncolo/oyaf404

**Published:** 2025-12-06

**Authors:** Saral Mehra, Sue S Yom, Barbara Burtness

**Affiliations:** Division of Otolaryngology, Department of Surgery, Yale School of Medicine, New Haven, CT 06520, USA; Department of Radiation Oncology, University of California San Francisco, San Francisco, CA 94143, USA; Section of Medical Oncology, Department of Medicine and Yale Cancer Center, Yale School of Medicine, New Haven, CT 06520, United States

**Keywords:** head and neck cancer, nivolumab, pembrolizumab

## Introduction

Two recent phase III trials, KEYNOTE (KN)-689^1^ and NIVOPOSTOP (GORTEC 2018-01)^2^ represent pivotal milestones in integration of PD-1 inhibition into curative-intent treatment of resectable head and neck squamous cell carcinoma (HNSCC). Both studies demonstrate event-free survival (EFS) benefits with immunotherapy, but they differ in design and patient selection. Taken together they establish a new standard of care for HPV-negative, PD-L1-expressing HNSCC managed with resection. They also raise critical questions about biology and trial design and will inform future studies of combination neoadjuvant regimens and of studies designed to bring immunotherapy to the curative chemoradiation setting.

Immune checkpoint inhibition (ICI) with PD-1 but not PD-L1 inhibitors^3^ or CTLA-4^4^ inhibitors has activity in recurrent/metastatic HNSCC and improves survival in the first-line setting^5^^,^^6^ as well as in patients previously treated with cisplatin.^7^^,^^8^ PD-L1 is the best-established biomarker for use of PD-1 inhibition in HNSCC. For patients treated on KN-048, the first-line trial of pembrolizumab or pembrolizumab plus chemotherapy compared to cetuximab plus chemotherapy, pembrolizumab was superior to cetuximab-chemotherapy for patients with CPS ≥1, and higher response rates and numerically longer survival were observed for pembrolizumab monotherapy for patients with CPS ≥ 20. Further dissection of PD-L1 status showed that pembrolizumab was numerically inferior to cetuximab-chemotherapy for patients with PD-L1 < 1.^9^ Real-world evidence examining the subgroup with CPS1-9 also shows activity in this subset, although outcomes appear superior when pembrolizumab is given with chemotherapy for this group.^10^

The activity of PD-1 inhibition in recurrent/metastatic HNSCC prompted several randomized trials to explore the impact of ICI in locally advanced HNSCC. The JAVELIN Head and Neck 100^11^ tested addition of the PD-L1 inhibitor avelumab (not validated in HNSCC) to chemoradiation (CRT) in locally advanced HNSCC, without PD-L1 biomarker selection. The study included patients with HPV-associated HNSCC that was deemed high risk, albeit these patients still have far lower event rates than those with HPV-negative cancers.^12^ The trial was negative, demonstrating a numerically worse outcome for patients treated with avelumab. The KN-412 trial similarly included patients with PD-L1-negative and HPV-associated HNSCC, here adding the validated PD-1 inhibitor pembrolizumab to radiation and concurrent high-dose cisplatin. The trial did not demonstrate a statistically significant advantage for addition of pembrolizumab, but subset analyses and long-term follow-up demonstrated numerically superior outcomes for patients with CPS ≥1 treated with pembrolizumab. With median follow-up of 74.4 months, median EFS was 70.9 months (95% CI 55.4-not reached [NR]) for pembrolizumab and 48.3 months (95% CI 26.8-66.8) for placebo (Hazard Ratio (HR) 0.80; 95% CI 0.64-0.98); median OS was NR for either arm, but an HR of 0.84 favored the pembrolizumab arm.^13^^,^^14^

Preclinical and clinical data substantiate the notion that tumor-draining lymph nodes play an important role in anti-tumor immune response to HNSCC and in antitumor effects of ICI, yet standard surgical and radiotherapy approaches ablate these nodes as potential sites of disease recurrence.^15^^,^^16^ Thus, there has been substantial interest in beginning immunotherapy while the tumor-draining lymph nodes are *in situ* and unirradiated. Phase II trials with pembrolizumab, nivolumab, or immunotherapy combinations^17–20^ given preoperatively consistently demonstrate pathologic evidence of tumor response, augmented immune response, and favorable outcomes. Given that PD-1 inhibition is active in recurrent/metastatic HNSCC and in the pre-operative setting for resectable disease, as well as indications from KN-412 that with adequate patient selection such an agent may also be given with chemoradiation, phase III testing in patients with resectable disease was in order.

## KN689

KN-689 was a peri-operative trial that compared standard-of-care (SOC) surgery followed by risk-adjusted adjuvant therapy using radiation with or without cisplatin to an experimental regimen of neoadjuvant pembrolizumab for 2 cycles followed by surgery and adjuvant therapy according to the same risk-adjusted guidelines, with the addition of pembrolizumab every 3 weeks for 45 weeks.^1^ Patients were enrolled across 3 geographies with 45% enrolled outside North America/European Union and only 16% from North America. All enrolled patients had *clinical* stage III/IV p16-negative squamous cell carcinoma of the oral cavity, larynx, hypopharynx, or oropharynx, or p16-positive oropharynx cancer that was T4N0-N2. It is notable that the majority had oral cavity cancer and only 4% were p16-positive, limiting generalizability to HPV-associated cancers. High-risk pathologic features requiring adjuvant chemotherapy and radiation included positive margin and extranodal extension. Randomization was stratified by PDL1 tumor proportion score ≥50% vs <50%, while the final analysis was performed using nested subgroup analysis based on combined positive score (CPS) of all comers, ≥1 (96%), and ≥10 (65%), with 5 cases (1%) missing a CPS score.

This study demonstrated a significant advantage for peri-operative pembrolizumab. For patients with CPS ≥ 10, median EFS was 59.7 months for pembrolizumab and 26.9 months for control (HR for progression, recurrence, or death, 0.66; 95% CI, 0.49-0.88; *P* = .004). In the entire group of PD-L1-expressing patients, median EFS was 59.7 months in the pembrolizumab group and 29.6 months for controls (HR 0.70; 95% CI, 0.55-0.89; *P* = .003). The primary endpoint was 3-year EFS rate. This was significantly increased in the CPS > 10, CPS > 1 and total populations. For the total population, 3-year EFS rates were 57.6% and 46.4%, respectively. There was no difference in ≥ Grade 3 adverse events. Overall survival results are not yet mature but also favor pembrolizumab (*P* = .02). Modest reduction in the intensity of post-operative therapy was reflected in an 11.6% reduction in the proportion of patients requiring cisplatin as part of risk-based post-operative therapy; the study did not quantify whether any reduction in surgical extent or reconstruction was achieved. Approximately 12% of patients in each arm did not undergo resection on trial, and surgical delays were seen in 10% of patients on pembrolizumab and 3% of control patients. Subset analysis indicated the most significant benefit for patients with Stage III disease at enrollment (HR 0.29).

These results indicate that neoadjuvant and adjuvant pembrolizumab added to resection and SOC post-operative therapy safely improve 3-year EFS over SOC, with some patients exhibiting major pathologic response (mPR) (9.4%) or complete response (3%). This regimen has been approved by the FDA and establishes a new standard of care in resectable, advanced stage mucosal HNSCC.

## NIVOPOST-OP

NIVOPOSTOP was a randomized, open-label phase III trial for patients with resected HNSCC, who had at least one pathologic feature indicating a high risk of recurrence.^2^ The trial was conducted by the Groupe d‘Oncologie Radiothérapie Tête et Cou (GORTEC) cooperative group, across 82 sites in 6 countries.

The study included patients age 18-75 years who had undergone macroscopic complete resection of a locally advanced SCC of the oral cavity, oropharynx, hypopharynx or larynx. Eligibility required pathologic stage III-IV, but stage II oropharyngeal cancer patients were eligible if they had pT3-4N1 and tobacco consumption of at least 20 pack-years. High-risk pathologic features were defined as extracapsular extension (ECE) of a lymph node, microscopically positive tumor margins (R1 resection or close margin ≤1 mm), ≥4 cervical nodes, or multiple foci of perineural invasion.

Patients were randomized 1:1 to receive postoperative radiation to 66 Gy with 3 cycles of concurrent cisplatin at 100 mg/m^2^ every 3 weeks (SOC), or the same chemoradiation (CRT) treatment with the addition of nivolumab. Randomization was stratified by p16 status and treatment center. Nivolumab 240 mg began 2 weeks before commencement of CRT, was continued at 360 mg every 3 weeks during CRT and then given at 480 mg every 4 weeks for 6 maintenance cycles. The primary endpoint was disease-free survival (DFS), powered to detect an HR of 0.65 favoring the nivolumab arm with a type I error rate of 0.05. There was a prespecified analysis analyzing DFS by PD-L1 expression status.

The analysis included 666 patients at a median follow-up of 30.3 months. Only 5% of the patients had p16-positive oropharynx cancer, again precluding extension of trial results to patients with HPV-associated HNSCC. About 25% had laryngeal or hypopharyngeal cancer. The 3-year DFS increased from 52.5% (95% CI, 46.2-58.4%) in the control arm to 63.1% (95% CI, 57-68.7%) with nivolumab. The DFS advantage appeared to result from a reduction in patients experiencing locoregional relapse. Nivolumab did not adversely affect completion of radiation or cisplatin. Grade 4 adverse events in the first 100 days after CRT occurred in 5.6% and 13.1% in the SOC and nivolumab arms; rates at 9 months were 0% and 1.2%. Two treatment-related deaths occurred in each arm. Overall survival (OS) results are not yet mature. Benefit from nivolumab was observed in patients with a positive PD-L1 CPS score. In subgroup analyses, there was a suggestion of a higher benefit from nivolumab among the 10% of patients without positive margin or ECE.

## Comparing the 2 trials

Both trials included only patients with locally advanced, resectable HNSCC. In KN-689, eligibility was determined on clinical features, with high-risk pathologic features present in 44% of patients in the control group and 32.5% in the pembrolizumab group. However, disparate reasons for drop out between the experimental and control arms of KN-689 could have been confounding. In contrast, in NIVOPOSTOP, a narrower group was enrolled: all patients had high-risk pathologic features with positive margin rates of 55%-60% and ECE in 62%-64% of patients. Patients were further selected by rapid recovery from resection and ability to begin nivolumab within 8 weeks and post-operative chemoradiotherapy within 10 weeks of resection (Table 1).

**Table 1.** Comparison of trial features for KEYNOTE-689 and NIVOPOSTOP studies.

	KN-689 (KEYNOTE-689)	NIVOPOSTOP
**Goal**	Improve outcomes by priming immune system before surgery.	Improve outcomes after resection in patients with high risk of recurrence pathologic features.
**Drug**	Pembrolizumab (anti-PD-1)	Nivolumab (anti-PD-1)
**Funding**	Merck	Bristol Myers Squibb
**Immunotherapy timing**	Both before and after surgery	Only after surgery
**Cancer type**	Locally advanced HNSCC	High-risk locally advanced HNSCC s/p surgery
**Setting**	**192 sites** European Union 40% (*n* = 283) North America 16% (*n* = 113) Rest of World 45% (*n* = 318)	**82 sites** European Union 100% (*n* = 666)
**Study group**	Neoadjuvant pembrolizumab → Surgery → RT (+/– cis) + pembrolizumab	Surgery → CRT + nivolumab
**Control group**	Surgery + RT (+/– cis)	Surgery + CRT
**Primary endpoint**	EFS (event-free survival)	DFS (disease-free survival)
**Secondary endpoints**	OS, pCR, safety	OS, LR failure, distant mets, death without previous disease failure, second primary malignancies, safety
**Key inclusion**	≥18 years ECOG performance status 0-1 clinical Stage III/IV oral cavity, larynx, hypopharynx, p16 neg oropharynx p16+ oropharynx T4N0-2	18-75 years ECOG performance status 0-1 pathologic Stage III/IV oral cavity, larynx, hypopharynx, oropharynx p16+ OPSCC if pT3N1 or pT4N1 and tobacco ≥ 20 pack-year Complete macroscopic resection.
**High-risk pathologic features**	ENE Positive margins or <1 mm	ECE Positive margins or <1mm Multiple PNI >4nodes (with or without ECE)
**Adjuvant therapy**	Standard of Care Low-Risk Group IMRT 60 Gy Standard of care High-Risk Group IMRT 66 Gy + cisplatin q3 weeks for 3 cycles Experimental Group Pembrolizumab 200 mg q3 weeks with RT or CRT for 3 cycles + 12 weeks cycles of adjuvant pembrolizumab Q3W Notes Those who did not undergo surgery (or who had R1 resection) could receive CCRT 70Gy + cisplatin q 3 weeks × 2 with or without pembrolizumab on the basis of trial group assignment).	Standard of Care IMRT 66 Gy in 33 fractions + cisplatin 100 mg/m^2^ every 3 weeks for 3 cycles (cisplatin-RT group). Experimental Group First administration of nivolumab 240 mg a minimum of 2 weeks after surgery Standard-of-care cisplatin-RT (starting 14 days (no less than 10 days) after the first nivolumab administration) with 3 cycles of concomitant nivolumab 360 mg Q3W (+/– 3 days) 6 cycles adjuvant nivolumab 480 mg Q4W (+/– 5 days) Notes Patients had to start radiotherapy and concomitant cisplatin within 4 to 10 weeks after surgery.
**Patients**	Analyzed 714 patients **CPS Score with 22C3 antibody** Negative 4% (*n* = 27) CPS ≥1 96% (*n* = 682) CPS ≥10 65% (*n* = 465) Missing 1% (*n* = 5) OPC P16 + 4% (*n* = 27)	Analyzed 666 patients **CPS Score with 28-8 antibody** Negative 12% (n = 78) CPS 1-19 45% (n = 298) CPS ≥20 37% (n-246) Not evaluated 7% (n = 44) OPC p16 + 5% (n = 33)
**Primary outcome**	**3-yr EFS** 57.6% vs. 46.4%, CPS ≥1: 58.2% vs 44.9%, CPS ≥10: 59.8% vs 45.9%	**3-yr DFS** 63.1% vs 52.5% Independent of PD-L1 status
**Select secondary outcomes**	**Adverse events** >3 AE: 44.6% in pembro group vs 42.9% **Major pathologic response** 9.4% All CPS: 34 patients CPS >1: 34 patients CPS >10: 32 patients **Complete response** 3% **OS** Estimated 3-year OS 68.4% vs. 61.1%, not yet mature for statistical testing	**Adverse events** First 100 days ≥3 AE 67.3 % cis + RT vs 72.4% nivo+cis + RT First 100 days Serious ≥3 AE 15.7% cis + RT. vs 26.9% nivo + cis + RT >100 days to 9 months ≥3 AE 4.9 % cis + RT. vs 11.3% nivo + cis + RT >100 days to 9 months Serious ≥3 AE 0% cis + RT vs 3.1% nivo + cis + RT Grade 4 Adverse events (9.3% vs 5.2%) in first 100 days. Then no different from 101 days to 9 months. **OS** Not yet available for analysis **Locoregional relapse** 15% of all patients (40% of events)

## Points of convergence and divergence

- Shared findings: Both trials confirm that integrating PD-1 blockade into curative-intent multimodality therapy is feasible and improves outcomes.
- Both highlight PD-L1 expression as a putative biomarker of benefit.
- KN-689 incorporates *perioperative immune priming*, while NIVOPOSTOP restricts to *adjuvant consolidation* in high-risk patients.
- Both studies included complete surgical dissection and postoperative radiation therapy to the draining lymph node basins.
- KN-689 spans a broader risk group (including intermediate-risk disease) with eligibility determined pre-operatively, whereas NIVOPOSTOP is restricted to patients having *high-risk* features.
- Both trials reported acceptable safety profiles without prohibitive increases in surgical/post-operative morbidity or adjuvant treatment delivery compromise, though treatment discontinuations due to immune-related adverse events were non-negligible.

The approach taken in KN-689 allows for treatment de-intensification in some responding patients. In KN-689, 9.3% of patients had mPR, defined as <10% of viable tumor cells in specimen, and 3% had a complete pathologic response. Most (32 of 34) patients with mPR were in the CPS ≥10 group. Furthermore, the significant reduction in high-risk pathologic features between the neoadjuvant vs SOC groups allows chemotherapy-sparing for some patients.

## Implications for biology and trial design

Several questions remain unanswered in the wake of the KN-689 and NIVOPOSTOP trials. Some examples include the following:

- What is the role and/or optimal duration of adjuvant immunotherapy?
- For patients with bulky disease, is there a role for addition of chemotherapy or novel agents to raise the response rate and/or reduce the risk of progression and unresectability?
- Can these results be replicated in a high-volume North American cohort, or in populations outside the United States and Europe?
- Is it correct to reduce the intensity of adjuvant therapy in responders, or should baseline characteristics and biomarkers also be considered?
- Should this approach also be adequately tested in high-risk HPV-related HNSCC, and in larynx and hypopharynx cancers?
- Finally, 8% of patients (*n* = 30) did not undergo further treatment after neoadjuvant pembrolizumab, including 15 patients with progressive disease: what were the causes and could this group be further analyzed in the real-world setting?

## Conclusion

Taken together, NIVOPOSTOP and KN-689 establish PD-1 blockade as a new standard component in curative-intent therapy for resectable HNSCC. However, the differing populations, designs, and outcomes complicate immediate application in clinical practice. Although the eligibility for the NIVOPOSTOP platform may be more familiar to many oncologists as it builds on accepted pathologic features to determine the intensity of the postoperative treatment, use of ICI only in the post-operative setting will deprive some patients of the benefits of ICI. In the NIVOPOSTOP framework, there will be no opportunity for a patient who started with high-risk disease to achieve sufficient response to omit cisplatin chemotherapy from the post-operative regimen; stage III patients without high risk features—who received substantial benefit from ICI in KN-689—will be deprived of the EFS and survival benefits of ICI therapy; and if, as preclinical data suggest, immunotherapy administration prior to surgical or radiotherapy ablation of tumor-draining lymph nodes offers the most robust augmentation of anti-tumor immune response, this opportunity is lost. An OS effect appears to be emerging for KN-689 while these data are less mature for NIVOPOSTOP. Thus, for most patients, pre-operative administration of PD-1 inhibition as in KN-689 will be preferred.

However, a small number of patients on the experimental arm of KN-689 were unable to proceed to resection. It will be critically important to understand the make-up of this subgroup in terms of PD-L1 expression, anatomic subsite, and tumor volume. For cases where the anatomical configuration or risk of progression pose a genuine risk to operability, immediate surgery is likely to be preferred by the surgeon and patient. In such a case, if pathologic high-risk features were identified, these would then dictate use of the NIVOPOSTOP regimen.

Future research for this group of patients could be focused on neoadjuvant regimens with higher response rates aimed at minimizing drop-out rates before resection, through addition of chemotherapy, targeted therapy or inhibitors of additional immune checkpoints. We also see an opportunity for more detailed exploration of dosing, length, expense, and toxicity of administration schedules to reduce burden on patients and the healthcare system.

## Data Availability

Not applicable.
